# Research on Pneumatic Proportional Pressure Valve Based on Silicon Microfluidic Chip with V-Shaped Electrothermal Microactuator

**DOI:** 10.3390/mi16050566

**Published:** 2025-05-08

**Authors:** Jun Zhang, Chengjie Zhou, Yangfang Wu

**Affiliations:** 1 School of Engineering, Hangzhou City University, Hangzhou 310015, China; zhangjun@hzcu.edu.cn; 2 School of Intelligent Manufacturing, Taizhou University, Taizhou 318000, China; 15167183166@163.com

**Keywords:** silicon microfluidic chip, pneumatic proportional pressure valve, V-shaped electrothermal microactuator, COMSOL Multiphysics, AMESim

## Abstract

This study presents a pneumatic proportional pressure valve employing a silicon microfluidic chip (SMC) integrated with a V-shaped electrothermal microactuator, aiming to address the limitations of traditional solenoid-based valves in miniaturization and high-precision control. The SMC, fabricated via MEMS technology, leverages the thermal expansion of microactuator ribs to regulate pressure through adjustable orifices. A first-order transfer function between input voltage and displacement of the microactuator was derived through theoretical modeling and validated via COMSOL Multiphysics 5.2a simulations. Key geometric parameters of the actuator ribs—cross-section, number, inclination angle, width, span length and thickness—were analyzed for their influence on lever mechanism displacement, actuator displacement, static gain and time constant. AMESim 16.0-based simulations of single- and dual-chip valve structures revealed that increasing ζ shortens step-response rise time, while reducing τ improves hysteresis. Experimental validation confirmed the valve’s static and dynamic performance, achieving a step-response rise time of <40 ms, linearity within the 30–60% input voltage range, and effective tracking of sinusoidal control signals up to 8 Hz with a maximum pressure deviation of 0.015 MPa. The work underscores the potential of MEMS-based actuators in advancing compact pneumatic systems, offering a viable alternative to conventional solenoids. Key innovations include geometry-driven actuator optimization and dual-chip integration, providing insights into high-precision, low-cost pneumatic control solutions.

## 1. Introduction

Pneumatic proportional pressure valves are indispensable in modern industrial automation, providing precise control over pressure, velocity, and force in applications such as robotic actuation, precision assembly, and medical devices [1,2,3]. Traditional proportional valves rely on electromagnetic solenoids to convert electrical signals into mechanical displacements, which regulate fluid flow by adjusting orifice areas. While these solenoid-driven systems are mature and reliable, their inherent limitations—including large physical footprints, high manufacturing costs, and limited precision in low-pressure regimes—have spurred interest in alternative actuation mechanisms [4,5]. The emergence of micro-electro-mechanical systems (MEMSs) has revolutionized fluidic control by enabling the fabrication of miniaturized valves with sub-millimeter dimensions and robust pressure handling capabilities [6,7,8]. Among these, silicon microfluidic chips stand out due to their compatibility with semiconductor manufacturing processes, allowing seamless integration with electronic circuits and sensors [9,10,11]. However, despite their potential, the application of SMC in dynamic pneumatic systems remains underexplored, particularly in scenarios requiring rapid response, low hysteresis, and high linearity. This gap motivates the present study, which seeks to bridge MEMS innovation with pneumatic control theory by developing a proportional pressure valve based on a V-shaped electrothermal microactuator embedded within an SMC.

The evolution of pneumatic proportional valves has been shaped by advancements in both actuation technologies and control strategies. Early designs, such as the nozzle-flapper mechanism proposed by Araki et al. [12], focused on improving linearity and reducing hysteresis in solenoid-driven systems. Subsequent efforts explored non-electromagnetic actuation principles to overcome limitations in power efficiency and miniaturization. For example, piezoelectric actuators, as demonstrated by Bao et al. [13], enabled high-stability pressure control with a steady-state accuracy of 0.25 kPa, while shape-memory alloys were utilized by Gradin et al. [14] to achieve large flow rates (3000 mL/min) in gas microvalves. Concurrently, MEMS-based microvalves diversified into biomedical and chemical applications, leveraging materials such as hydrogels [15] and magnetic fluids [16] for pH- or field-responsive flow regulation. Silicon microfluidic chips, a subset of MEMS valves, emerged as a promising platform due to their mechanical robustness and scalability. Vandelli et al. [17] pioneered early SMC prototypes, demonstrating leakage-resistant operation up to 1.4 MPa, while later studies optimized structural layers to mitigate fatigue [18]. Despite these achievements, SMC remains predominantly confined to HVAC systems [19], with limited adoption in pneumatic control. A critical barrier lies in the dynamic performance of SMC actuators, particularly V-shaped electrothermal microactuators, which exhibit complex electro-thermo-mechanical coupling. While foundational models by Hussein et al. [20] and Lott et al. [21] elucidated static behaviors, transient characteristics—such as step response and frequency-dependent hysteresis—remain poorly understood. Furthermore, geometric parameter optimization, essential for tailoring actuator performance to specific applications, has received scant attention. For instance, prior works by Chen et al. [22] and Hoang et al. [23] highlighted the influence of rib cross-sections on displacement output but neglected the interplay between multiple geometric factors (e.g., inclination angle, width, and length) and their collective impact on system-level valve performance.

This study addresses these gaps by developing a MEMS-based pneumatic proportional pressure valve centered on a silicon microfluidic chip with a V-shaped electrothermal microactuator. The research encompasses four main phases: (1) establishing a dynamic model for the V-shaped electrothermal microactuator, establishing a first-order transfer function between input voltage and displacement; (2) optimizing valve performance via parametric analysis of rib geometry using COMSOL Multiphysics 5.2a simulations, with emphasis on enhancing static gain (*ζ*) and reducing time constant (*τ*); (3) validating system-level functionality through AMESim 16.0-based simulations and experimental tests, focusing on hysteresis mitigation, step response, and linearity under varying frequencies; (4) **experimental validation** of the valve’s static and dynamic performance, including step response, linearity, and frequency-dependent hysteresis. The work innovatively integrates geometry-driven actuator design—analyzing the effects of rib cross-section, count, inclination angle, width, and length on static gain (*ζ*) and time constant (*τ*)—with dual-chip parallelization to balance miniaturization and flow capacity. By bridging MEMS fabrication with pneumatic control theory, this research provides a framework for developing compact, high-precision proportional valves, offering insights into the application of thermal actuators in dynamic fluidic systems.

## 2. Modeling and Simulation of V-Shaped Electrothermal Microactuator

### 2.1. Structure of the Silicon Microfluidic Chip

**Figure 1:**
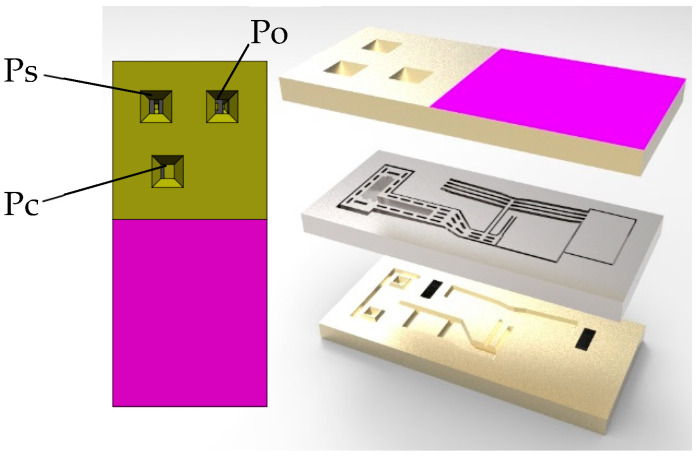
Structure diagram of the silicon microfluidic chip.

**Figure 2:**
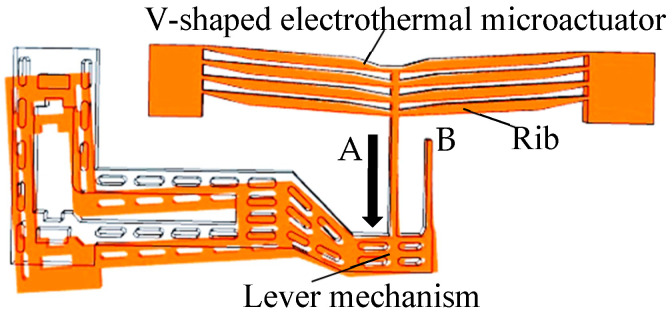
Structure diagram of the actuator in the middle layer.

The structure of the microfluidic chip is shown in Figure 1, with geometric specifications as follows: a length of 10.8 mm, a width of 4.83 mm, a height o 2.225 mm, and a three-layer design. The Ps port serves as the input, the Pc port as the controlled output, and the Po port as the feedback. Under zero-voltage input, the Ps port remains normally closed, while the Po port is normally open. The actuator in the middle layer of the chip (Figure 2) consists of a V-shaped electrothermal microactuator (with four pairs of ribs) and a lever mechanism.

The movable part of the middle layer functions as the valve core. When a control voltage is applied, Joule heating occurs in the V-shaped electrothermal microactuator, raising the temperature of the ribs and inducing thermal expansion. This generates displacement along direction A (Figure 2). Point B acts as the fulcrum of the lever mechanism to amplify the displacement, thereby adjusting the flow areas of the Ps and Po ports to regulate output pressure or flow rate.

**Figure 3:**
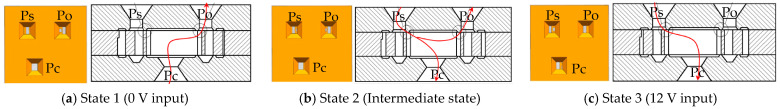
Operational states of the ports (Ps, Po, and Pc) in the silicon microfluidic chip. The red arrows in the figure indicate gas flow directions.

The operational states of the ports (Ps, Po, and Pc) in the silicon microfluidic chip across three working conditions are illustrated in Figure 3.

State 1 (0 V Input): As shown in Figure 3a, the Ps port remains closed in the initial state, whereas the Po and Pc ports are fully open and interconnected.

State 2 (Intermediate state): In Figure 3b, the flow area of the Ps port gradually increases with rising input voltage, while the Po port’s flow area decreases. Gas flows from the Ps port to both the Pc and Po ports.

State 3 (12 V Input): At the maximum rated voltage (Figure 3c), the Po port is fully closed, and the Ps port connects directly to the Pc port, achieving full opening.

### 2.2. Structure and Material Properties of V-Shaped Electrothermal Microactuator

**Figure 4:**
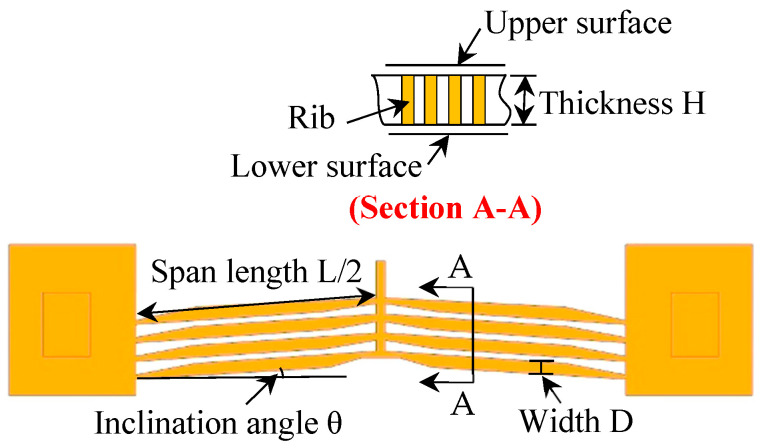
Structure diagram of the V-shaped electrothermal microactuator.

The V-shaped electrothermal microactuator, a core component of the silicon microfluidic chip, consists of four pairs of symmetrically arranged silicon ribs with variable cross-sections (Figure 4). The actuator is fabricated from single-crystal silicon, chosen for its high tensile strength and controllable resistivity [24]. Partial material properties and geometric parameters of the V-shaped electrothermal microactuator are listed in Table 1.

### 2.3. Theoretical Modeling

**Figure 5:**
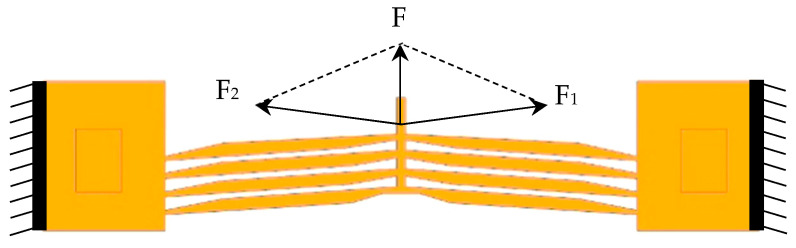
Force diagram of the actuator during thermal expansion.

The actuator’s displacement arises from Joule heating-induced thermal expansion. When current flows through the actuator, the thermal expansion of the ribs generates the actuation effect as shown in Figure 5. The resultant force *F* at the rib ends is calculated as follows [18]:

(1) F=2EAh⋅ΔT⋅sinθ

where *E* is the Young’s modulus of the material (GPa), *A* is the cross-sectional area of the ribs (mm^2^), *α* is the coefficient of thermal expansion (K^−1^), Δ*T* is the temperature difference (K), and *θ* is the inclination angle of the ribs (°).

By modeling the V-shaped electrothermal microactuator as a mass–spring–damper system, Newton’s second law gives the following:

(2) $$ma=F_{s}+F_{f}+F_{r}+F$$

where *m* is the mass (kg), *a* is the equivalent acceleration (m·s^−2^), *F*_s_ is equivalent spring force (N), *F*_f_ is equivalent frictional force (N), and *F*_r_ is equivalent viscous force (N).

The calculation formula for equivalent spring force is as follows:

(3) $$F_{s}=-k_{s}\cdot x_{0}$$

where *k*_s_ is the equivalent spring stiffness (N·mm^−1^), and *x*_0_ is the output displacement of the actuator (mm).

The formula for calculating the equivalent viscous force experienced by the rib during movement is as follows:

(4) $$F_{r}=-\beta \cdot x_{0}^{\prime }$$

where *β* is the equivalent damping coefficient.

The resultant force *F* is expressed as a function of the control voltage *U*:

(5) F=αU

where *α* is the proportionality coefficient.

As shown in Figure 4, the ribs are not in contact with the upper or lower surfaces, resulting in zero equivalent friction (*F*_f_ = 0). Additionally, due to the extremely small mass (*m* ≈ 0), the “*ma*” term is negligible. Equation (2) simplifies to the following:

(6) $$\beta x_{0}^{\prime }+k_{s}x_{0}=\alpha U$$

Applying the Laplace transform, the transfer function between input voltage and actuator output displacement is derived as follows:

(7) $$\frac{X_{0}\left(S\right)}{U\left(S\right)}=\frac{\alpha }{\beta S+k_{s}}=\frac{\zeta }{1+\tau S}$$

where *ζ* is the static gain coefficient (*ζ* = *α*/*k*_s_), and *τ* is the time constant (*τ* = *β*/*k*_s_).

The output displacement of the lever mechanism is proportional to the actuator displacement, and its transfer function with respect to input voltage is also represented by Equation (7).

### 2.4. COMSOL Multiphysics Simulation of the V-Shaped Electrothermal Microactuator

COMSOL Multiphysics software was utilized to perform multiphysics simulations of the V-shaped electrothermal microactuator in the microfluidic chip, involving coupled thermal, electrical, and structural mechanics fields [25].

The simulation model of the chip actuator incorporated the Structural Mechanics Module and Heat Transfer Module in COMSOL Multiphysics. The Structural Mechanics Module includes integrated submodules for coupled electro-thermo-mechanical fields, with boundary conditions such as voltage, temperature, and fixed constraints. These features are suitable for most studies involving electro-thermo-mechanical coupling.

**Figure 6:**
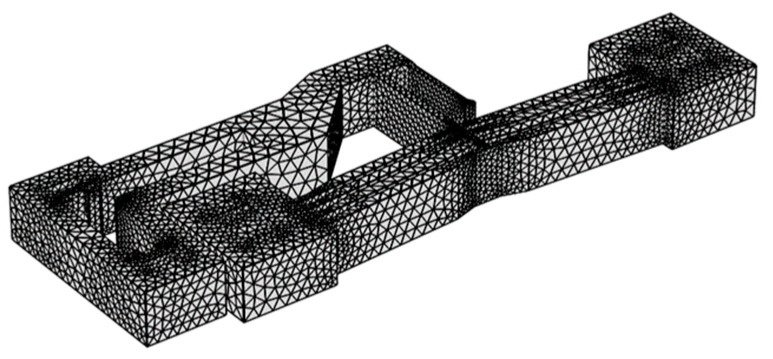
Mesh generation.

**Figure 7:**
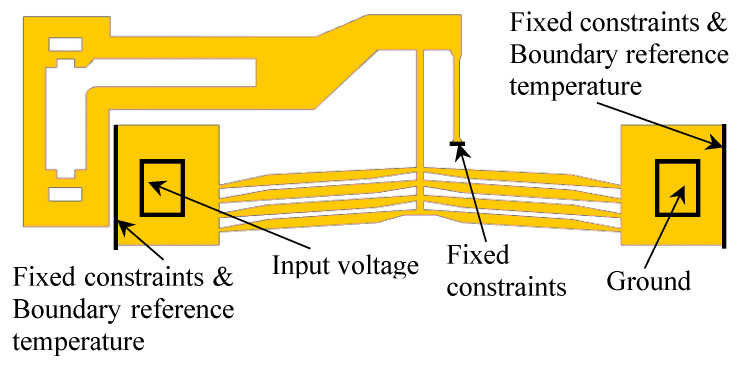
Boundary conditions.

The meshing of the actuator with tetrahedral elements and the applied boundary conditions are illustrated in Figure 6 and Figure 7, respectively. Experimental studies indicate that an input control voltage of 8V generates moderate displacement in the lever mechanism [18]; thus, 8 V was selected as the reference input voltage for simulation.

**Figure 8:**
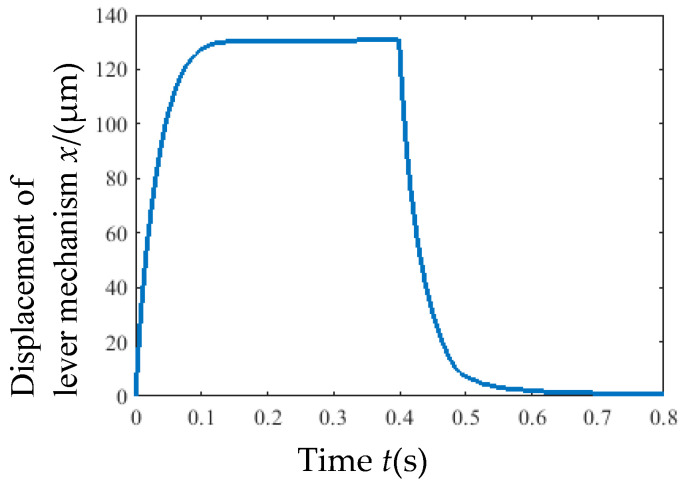
The curve of the displacement of the lever mechanism with time.

**Figure 9:**
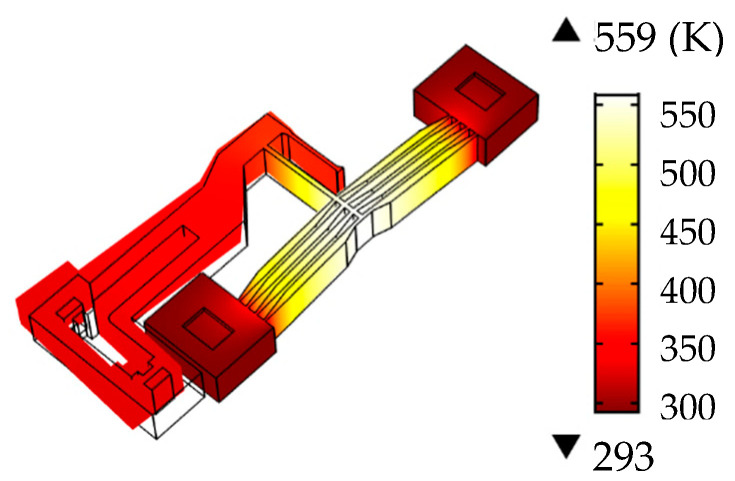
The temperature distribution of the ribs of the actuator.

A simulation analysis was conducted using an 8 V step signal as the input voltage, and the time-dependent curve of the output displacement *x* of the lever mechanism is illustrated in Figure 8. At *t* = 0.4 s, the actuator reached a steady state; the temperature distribution on the ribs is illustrated in Figure 9. Simulation results indicate that the maximum displacement of the lever mechanism was approximately 131.6 μm under 8 V input, and the highest temperature (559 K) occurred at the central region of the ribs in a steady state.

### 2.5. Analysis of Geometric Parameter Effects on the Actuator

According to Equation (7), the static gain coefficient (*ζ*) and time constant (*τ*) in the actuator’s transfer function depend on the geometric parameters of the ribs. This section employs the Multiphysics simulation model to analyze the effects of rib parameters (cross-section, number, inclination angle, width, span length, and thickness) on the lever mechanism displacement *x*, actuator displacement *x*_0_, static gain *ζ*, and time constant *τ*.

#### 2.5.1. Influence of Rib Cross-Section and Number Variation

**Figure 10:**
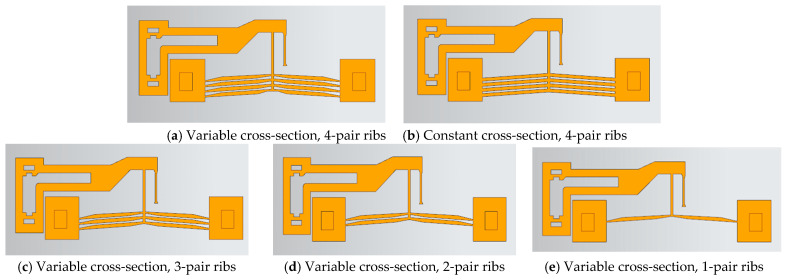
Actuator structures with variable cross-section and the number of ribs.

With the rib inclination angle fixed, the cross-section area and number of ribs were varied. The geometric configurations are shown in Figure 10a–e.

**Figure 11:**
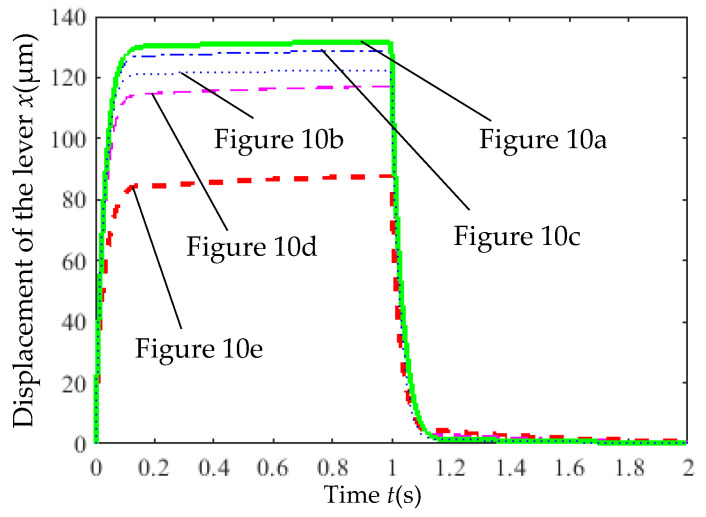
The influence of the variation in the cross-section and the number of ribs on the displacement of the lever.

Figure 11 shows the influence of cross-section and rib number variations on the lever displacement under an 8 V step voltage input, while Table 2 lists the corresponding actuator displacements. Figure 11 indicates that the time for the lever displacement to transition from 10% to 90% of the steady-state value was less than 0.07 s, suggesting minimal impact of cross-section and rib number on response time. When the control voltage was reset to 0 V, the lever did not fully return to its initial position, with four-pair rib configurations exhibiting better recovery than single-pair configurations, likely due to structural mechanical effects. Table 2 reveals a lever displacement amplification factor of approximately 11.3. The actuator with four pairs of variable cross-section ribs produced a displacement of 11.7 μm, representing a 51.9% increase compared to single-pair variable cross-section ribs (7.7 μm) and a 9% increase over four-pair constant cross-section ribs (10.7 μm), highlighting the significant impact of rib number on displacement.

**Figure 12:**
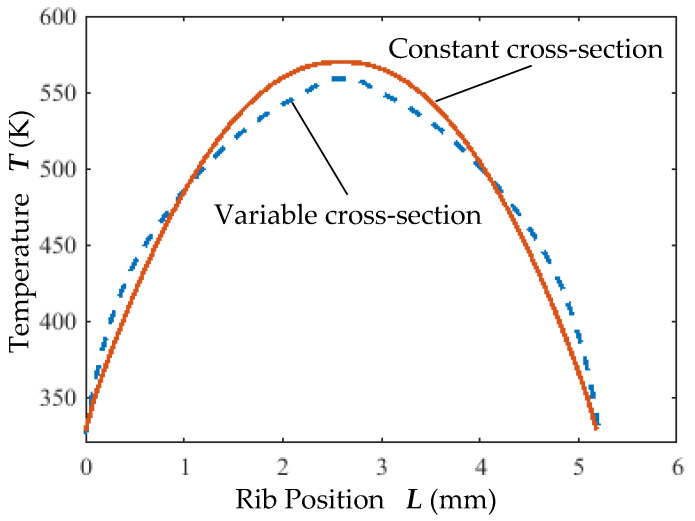
Effect of cross-section variation on rib temperature distribution.

The effect of cross-section variation on the temperature distribution of the ribs is shown in Figure 12. In a steady state, the highest temperature occurred at the central region of the ribs. For variable cross-section ribs, the maximum temperature was 559 K, whereas constant cross-section ribs reached 569.8 K. The average temperature rise of variable cross-section ribs (491 K) was lower than that of constant cross-section ribs (493 K). Under the same input voltage, variable cross-section ribs generated larger displacements with lower temperatures.

The effects of rib cross-section and number on the static gain coefficient (*ζ*) and time constant (*τ*) are summarized in Table 3 and Table 4, respectively. The static gain *ζ* is influenced by both cross-section and rib number, while the time constant *τ* is significantly affected by cross-section variation.

#### 2.5.2. Influence of Rib Inclination Angle

**Figure 13:**
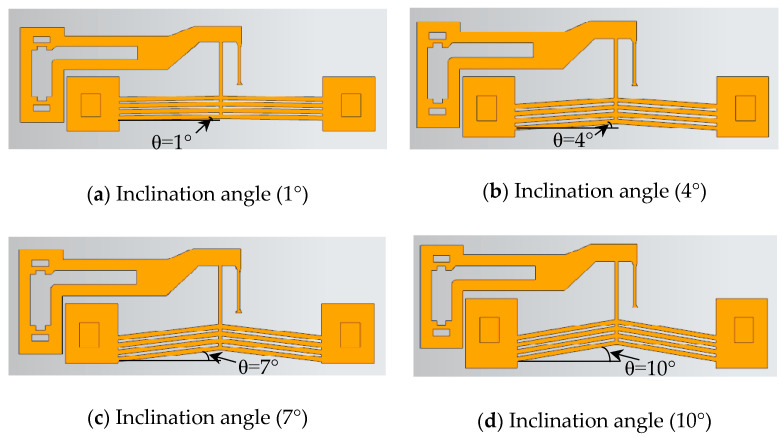
Actuator structures with different rib inclination angles.

With the rib number and cross-section fixed, the inclination angle (*θ* = 1°, 2°, 3°, 4°, 5°, 6°, 7°, 8°, 9°, 10°) was varied. The geometric configurations of the inclination angles of 1°, 4°, 7°, and 10° are shown in Figure 13. 

**Figure 14:**
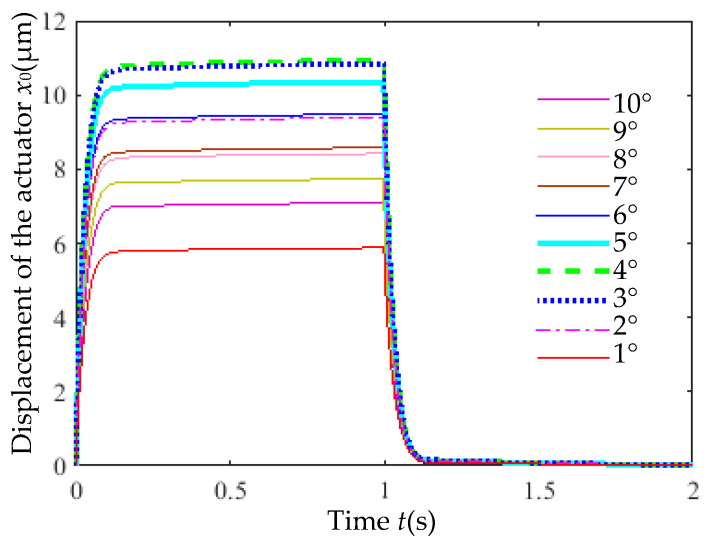
Output displacement of actuators with varying rib inclination angles versus time.

Figure 14 shows the output displacement (*x*_0_) of actuators with varying inclination angles with an 8 V step voltage input. The inclination angle had a minimal impact on response time (<0.07 s).

**Figure 15:**
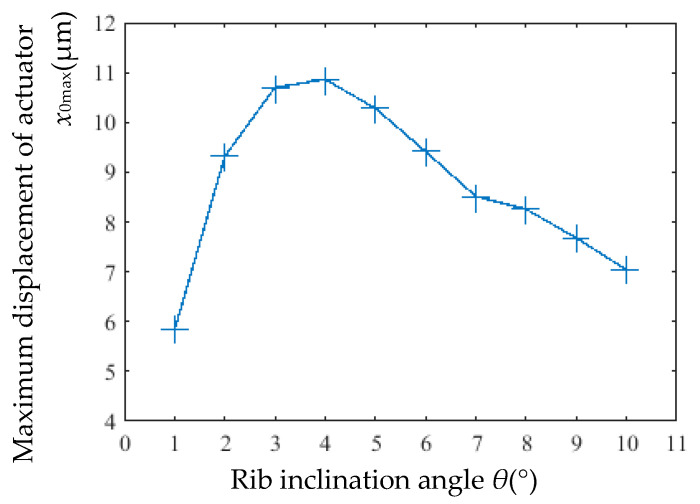
Maximum displacement versus rib inclination angle.

However, as shown in Figure 15, the maximum displacement (*x*_0max_) was significantly affected: at 8 V, *x*_0max_ initially increased and then decreased with larger inclination angles. The peak displacement of 10.86 μm occurred at 4°, while the minimum displacement of 5.83 μm was observed at 1°.

The static gain coefficient (*ζ*) and time constant (*τ*) for different rib inclination angles are listed in Table 5. The static gain is significantly influenced by the inclination angle. As the inclination angle increases, *ζ* first increases and then decreases. The minimum *ζ* occurs at 1°, while the maximum *ζ* is observed at 4°, which aligns with the trend of the actuator’s maximum output displacement (*x*_0max_) versus inclination angle.

#### 2.5.3. Influence of Rib Width, Span Length, and Thickness

**Figure 16:**
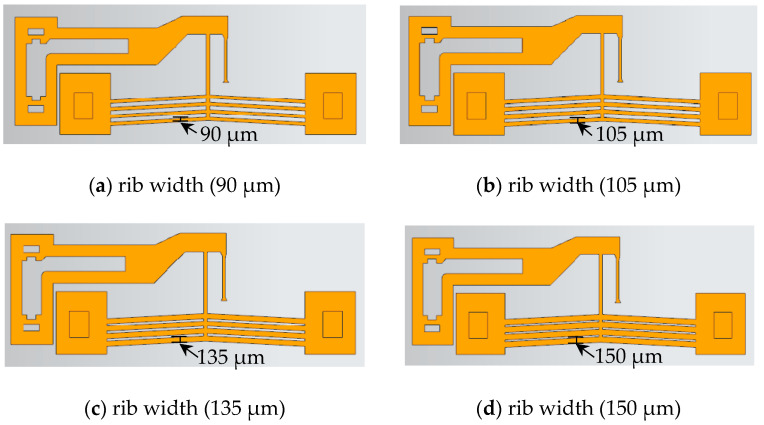
Actuator structures with different rib widths.

With the rib number and cross-section area fixed, the rib width (*D*) was adjusted to 0.75× (90 μm), 0.875× (105 μm), 1.125× (135 μm), and 1.25× (150 μm) of the initial width (120 μm). The geometric configurations are shown in Figure 16.

**Figure 17:**
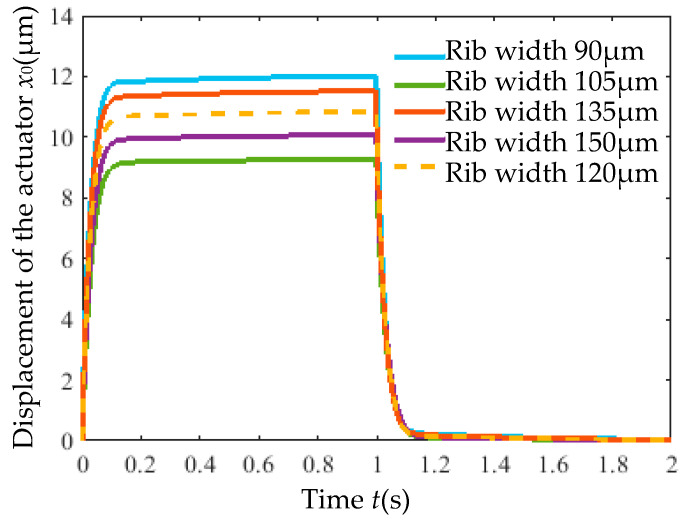
Output displacement of actuators with varying rib width versus time.

Figure 17 shows the displacement of actuators with varying rib widths under an 8 V step voltage. Reducing the rib width increased the output displacement but had a negligible impact on response time (<0.07 s). As listed in Table 6, rib width primarily influenced the static gain *ζ*, which decreased with increasing width.

**Figure 18:**
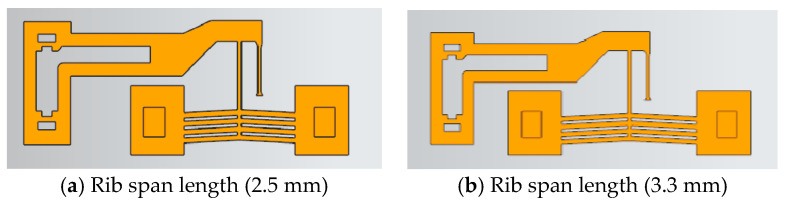
Actuator structures with different rib span lengths.

With other rib parameters fixed, the rib span length *L* was adjusted to 0.5× (2.5 mm) and 0.67× (3.3 mm) of the initial value. The geometric configurations are shown in Figure 18.

**Figure 19:**
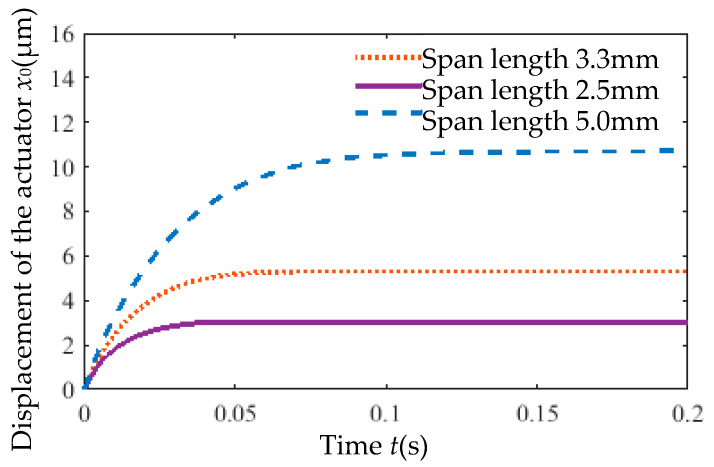
Output displacement of actuators with varying rib span lengths versus time.

Under an 8 V step voltage input, the output displacement of actuators with varying span lengths is shown in Figure 19. For span lengths of 0.5×, 0.67×, and 1× the original value, the time required for displacement to rise from 10% to 90% of the steady-state value was 0.026 s, 0.032 s, and 0.056 s, respectively. Doubling the span length increased the response time by approximately 1.15×, indicating a significant impact of span length on response time.

The effects of rib span length variation on the static gain coefficient (*ζ*) and time constant (*τ*) are summarized in Table 7. Both *ζ* and *τ* are significantly influenced by span length, with decreasing values observed as the span length is reduced.

**Figure 20:**
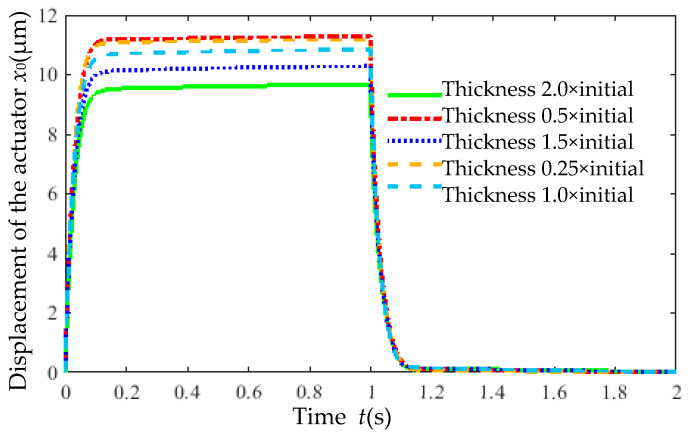
Output displacement of actuators with varying rib thicknesses versus time.

With other parameters fixed, the rib thickness *H* was adjusted to 0.25× (0.1875 mm), 0.5× (0.375 mm), 1.5× (1.125 mm), and 2× (1.50 mm) of the initial value (0.75 mm). The corresponding output displacement versus time is shown in Figure 20. Rib thickness had a minimal impact on response time. Reducing the thickness increased the output displacement, which stabilized when the thickness reached 0.375 mm. As listed in Table 8, rib thickness significantly affected the static gain *ζ*, which increased with decreasing thickness but stabilized below 0.375 mm.

**Figure 21:**
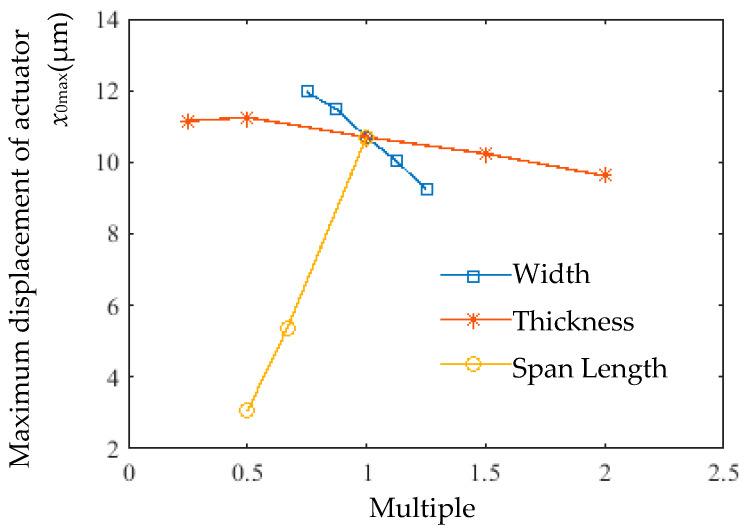
Effect of rib width, thickness, and span length on actuator output displacement.

The combined effects of rib width, thickness, and span length on actuator output displacement are summarized in Figure 21. Compared to width and thickness variations, span length had the most significant impact. The displacement of the actuator with the original span length (10.7 μm) was 3.5× that of the actuator with half the span length (3.055 μm).

## 3. Modeling and Simulation of Proportional Valve Using SMC

### 3.1. Working Principle of the Silicon Microfluidic Chip

**Figure 22:**
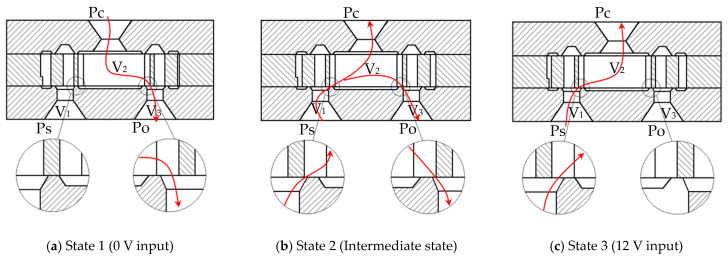
Working principle of the silicon microfluidic chip.

The working principle of the microfluidic chip is illustrated in Figure 22. Typically, chamber V_1_ is connected to the pneumatic source, chamber V_2_ serves as the controlled output, and chamber V_3_ is vented to ambient air. The red curves in the figure indicate gas flow directions. By applying a control voltage (0–12 V DC), the movable part of the middle-layer lever mechanism adjusts the flow areas of the variable ports Ps and Po, thereby proportionally regulating the output pressure or flow rate at port Pc.

State 1 (0 V input): As shown in Figure 22a, the lever mechanism is in its initial position. Port Ps is closed, while port Po is fully open, connecting chambers V_2_ and V_3_.

State 2 (Intermediate state): Increasing the control voltage enlarges the flow area of Ps and reduces that of Po (Figure 22b).

State 3 (12 V input): At maximum voltage, port Po is closed, and Ps is fully open, establishing a direct connection between chambers V_1_ and V_2_ (Figure 22c).

### 3.2. Structure of the Proportional Valve Using Silicon Microfluidic Chip

The microfluidic chip has a uniquely compact structure, with a maximum valve orifice flow area of 0.045 mm^2^. Its intrinsic pressure regulation capability provides advantages for low-pressure, high-precision control applications.

**Figure 23:**
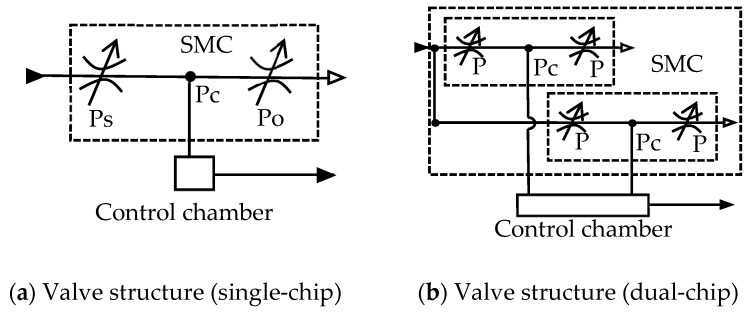
Schematic of the single-chip and dual-chip valve structure.

The schematic structures of the pneumatic proportional pressure valve with single-chip and dual-chip configurations are shown in Figure 23, respectively. Both configurations function as adjustable half-bridge circuits.

### 3.3. AMESim Modeling of Proportional Valve

AMESim is a multidisciplinary simulation software. It integrates foundational libraries for mechanical, electrical, hydraulic, pneumatic, and thermal domains [26].

**Figure 24:**
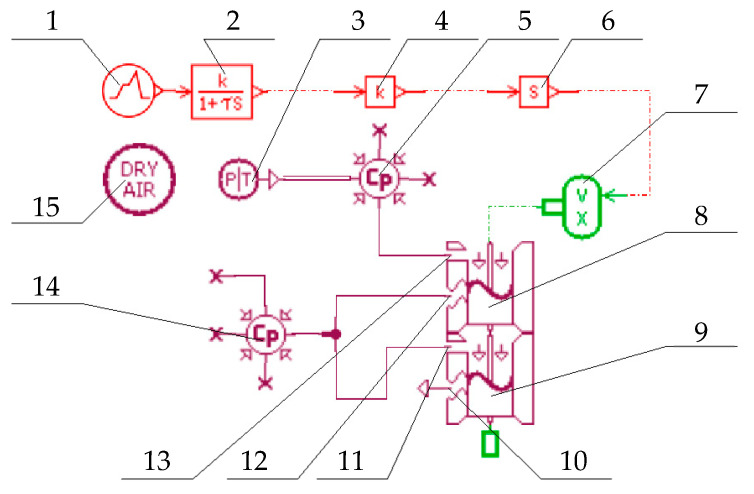
Pneumatic simulation model of the single-chip valve structure. 1 Signal generator; 2 First-order transfer function; 3 Ideal pneumatic source; 4 Gain; 5 Variable volume chamber; 6 Derivative; 7 Velocity-displacement converter; 8 Pneumatic spool with specific orifice; 9 Pneumatic spool with specific orifice; 10 Po port; 11 Pc port; 12 Pc port; 13 Ps port; 14 Variable volume chamber; 15 Gas properties of the source.

The pneumatic simulation model built using AMESim component libraries is shown in Figure 24. Based on the physical structure, and the working principles of the single-chip proportional valve, the simulation model was simplified into three main components: (1) the lever mechanism driven by the electrothermal actuator, (2) the Ps and Pc ports with flow areas modulated by the lever displacement, and (3) the control chamber in the base.

Components 1, 2, 4, 6, and 7: These components represent the moving part of the lever mechanism driven by the electrothermal microactuator. Component 1 is a signal generator providing the control voltage (0–12 V). Component 2 implements the first-order transfer function (Equation (7)) with *k* = 16.407 and *τ* = 0.032452 to convert voltage into displacement. Components 4, 6, and 7 translate numerical outputs into physical lever displacements.

Components 8 and 9: These components model the Ps and Pc ports as pneumatic spool valves with specific orifices, where the flow area varies with spool displacement.

Components 14 and 15: The control chamber is represented by Component 14, with a volume set to 200 mm^3^. Component 15 defines the working medium as dry air.

Component 3: This component is the ideal pneumatic source. The operating pressure is 0.7 MPa.

**Figure 25:**
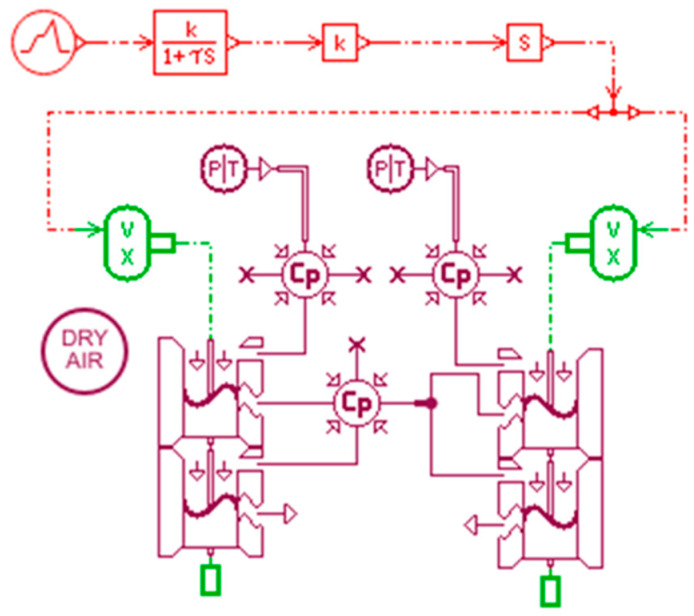
Pneumatic simulation model of the dual-chip valve structure.

The AMESim pneumatic simulation model of the dual-chip proportional valve is shown in Figure 25. It consists of two microfluidic chip models connected in parallel based on the single-chip structure.

### 3.4. Simulation of Closed-Loop Control System

The pneumatic proportional pressure valves with single-chip and dual-chip structures share similar configurations and working principles. The following simulation analysis focuses on the dual-chip valve.

#### 3.4.1. Closed-Loop Control System Simulation Model (Dual Chip)

**Figure 26:**
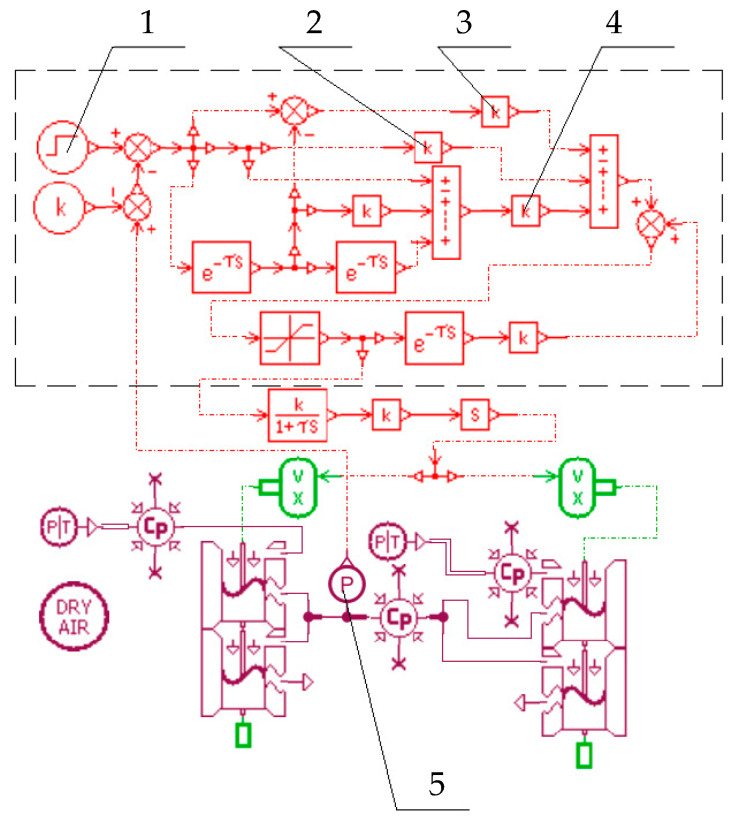
AMESim-based closed-loop control system simulation model. 1 Signal generator; 2 Gain (integral time constant *k*_i_); 3 Gain (proportional gain *k*_p_); 4 Gain (derivative time constant *k*_d_); 5 Pressure sensor.

The closed-loop control system employs the incremental PID algorithm, and its AMESim simulation model of the dual chip is shown in Figure 26.

In Figure 26, the incremental PID controller is enclosed within the black dashed box. Component 1 generates the input control voltage signal (0–5 V), corresponding to the proportional valve’s output pressure range of 0–0.5 MPa. Components 2, 3, and 4 represent the PID parameters ***k***_i_ (integral time constant), ***k***_p_ (proportional gain), and ***k***_d_ (derivative time constant), respectively. Component 5 is a pressure sensor that converts the pressure signal from the control chamber into an analog signal, transmits it to the controller, and calculates the error (***k***).

#### 3.4.2. Closed-Loop Characteristic Simulation Analysis (Dual-Chip)

(1) Step Response Characteristic

**Figure 27:**
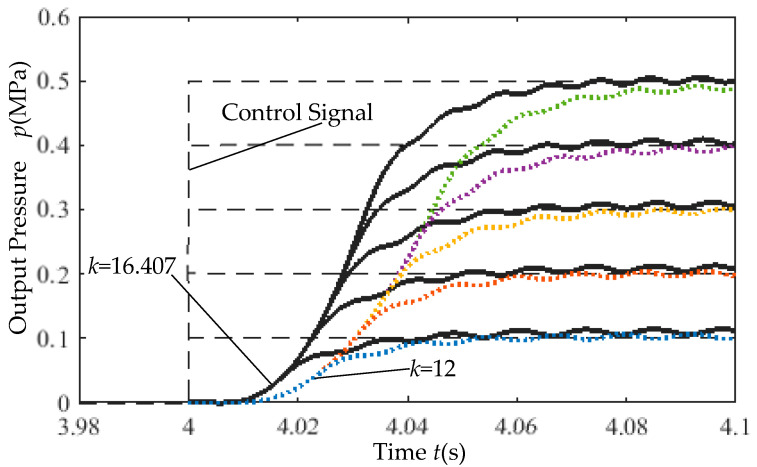
Step response simulation results.

With the pneumatic source pressure set to 0.7 MPa, the step response of the closed-loop control system using incremental PID is shown in Figure 27. The dashed line represents the reference control signal. Simulation results indicate that the dual-chip proportional valve achieves a step response rise time of less than 43 ms, demonstrating excellent responsiveness. A higher static gain coefficient reduces the rise time under the same input voltage.

(2) Input–Output Characteristic

**Figure 28:**
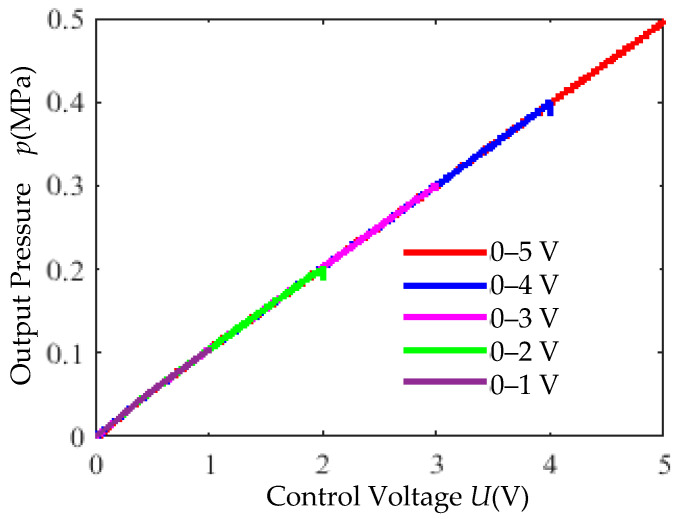
Input–output characteristic simulation curves.

Under a pneumatic source pressure of 0.7 MPa, triangular wave control signals (0–1 V, 0–2 V, 0–3 V, 0–4 V, 0–5 V) with a period of 4 s were applied. The input–output characteristic curves in Figure 28 demonstrate the high linearity of the control system.

(3) Sinusoidal Response Characteristic

**Figure 29:**
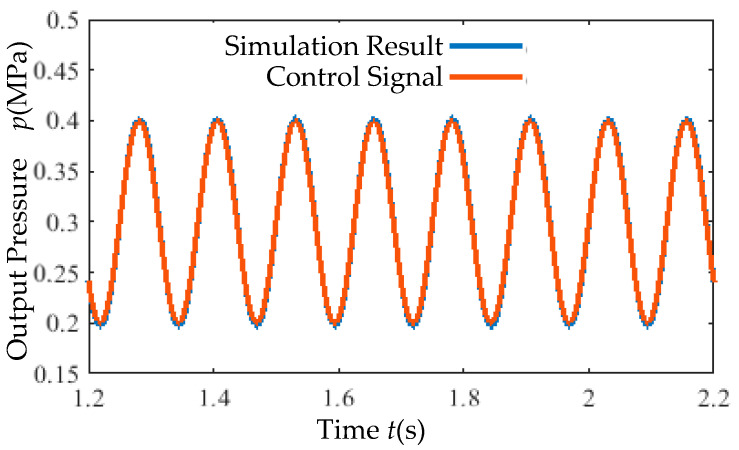
Sinusoidal response simulation curve.

Under an 8 Hz sinusoidal control signal, the simulation output (Figure 29) closely follows the input signal, indicating superior dynamic performance.

(4) Hysteresis Characteristic

**Figure 30:**
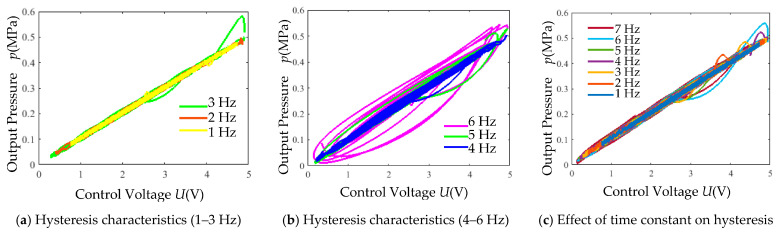
Simulation results of output pressure versus control voltage.

Exponential decay signals (5 V amplitude) with varying frequencies were applied. The simulated pressure outputs versus control voltage are shown in Figure 30.

When the input signal frequency exceeds 5 Hz, the irregular hysteresis loops of the output pressure gradually expand with increasing frequency. The original model in Figure 30a,b has a time constant *τ* = 0.03. By reducing *τ* to 0.01, the effect on hysteresis characteristics is illustrated in Figure 30c. Even under a 7 Hz exponential decay input signal, the modified model (*τ* = 0.01) exhibits improved hysteresis performance compared to the model with *τ* = 0.03. This demonstrates that decreasing *τ* effectively mitigates hysteresis effects.

## 4. Experimental Validation

The dual-chip pneumatic proportional pressure valve was selected as the experimental subject. The objectives were as follows: (1) to evaluate the valve’s performance through open-loop tests, step response tests, input–output characteristic tests, sinusoidal response tests, and hysteresis characteristic tests, and (2) to validate the simulation model by comparing experimental and simulated results.

### 4.1. Experimental System Construction

**Figure 31:**
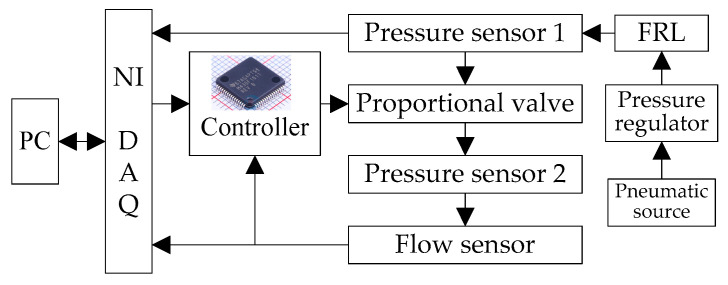
Schematic of the experimental system.

The experimental system for the proportional pressure valve with silicon microfluidic chip is illustrated in Figure 31.

**Figure 32:**
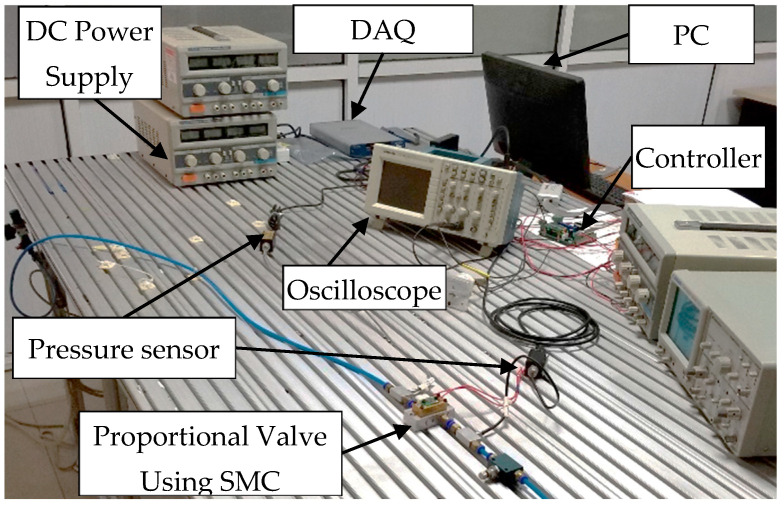
Physical platform of the experimental system.

It comprises a PC, a high-speed data acquisition card (DAQ), a controller, and pressure/flow sensors. The PC communicates with the DAQ and executes control signal generation and data acquisition via LabVIEW. The DAQ converts control signals into 0–5 V voltages for the controller and collects analog feedback from sensors. The controller employs an incremental PID algorithm to minimize the error between the control signal and pressure feedback, achieving closed-loop control. A high-pressure nitrogen cylinder (≈10 MPa) serves as the pneumatic source, regulated to 1 MPa via a pressure regulator and stabilized at 0.7 MPa using a filter–regulator–lubricator (FRL) unit. Pressure sensor 1 monitors the source pressure, while sensor 2 measures the valve output pressure for feedback. The experimental system’s physical platform is shown in Figure 32.

The key components and parameters of the system are as follows:

Pressure sensor: CYY4 miniature pressure sensor (Xi’an Weizheng Electronic Technology Co., LTD, Xi’an, China), range 0–1 MPa, output current 4–20 mA, accuracy class 0.25%.

Flow sensor: SFAB-200U flow sensor (FESTO, Esslingen, Germany), operating pressure 0–1 MPa, flow range 0–200 L/min, output current 4–20 mA.

Controller: Developed with TI MSP430F1611 microcontroller (Texas Instruments, Dallas, TX, USA), featuring 16-bit MCU, 46 kB Flash, 10 kB RAM, 12-bit ADC, and dual DAC.

DAQ: NI USB-6361 (National Instruments, Austin, TX, USA), sampling frequency 10 kHz, 16-bit analog input (2 MS/s), 2 analog output channels (2.86 MS/s).

Host Computer: A PC serves as the control host (DELL, Round Rock, TX, USA), communicating bidirectionally with the DAQ. A LabVIEW 2014-based GUI was developed for data acquisition and processing.

### 4.2. Experimental Results and Analysis

**Figure 33:**
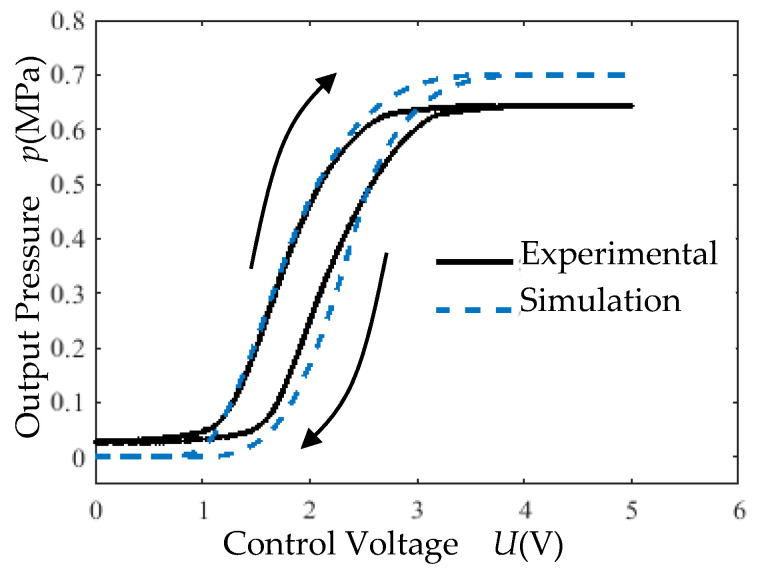
Triangular wave hysteresis experimental curve.

**Figure 34:**
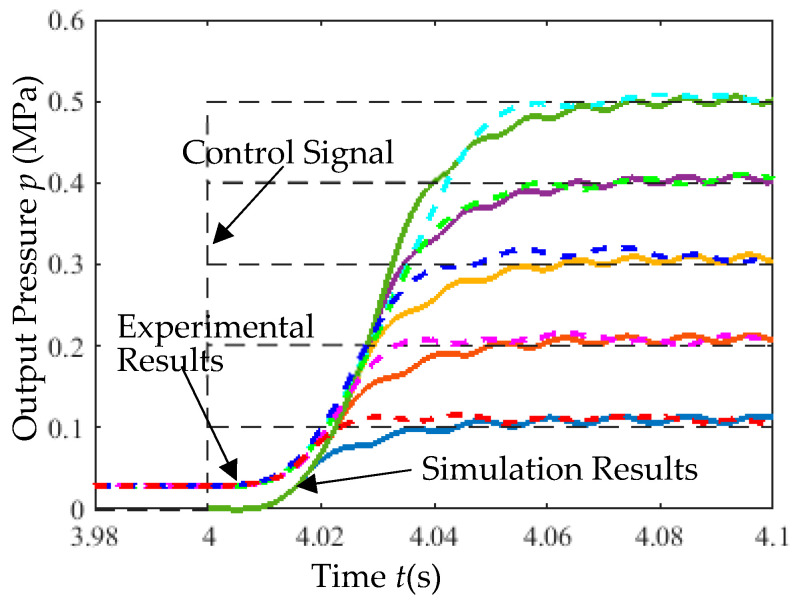
Step response experimental results.

**Figure 35:**
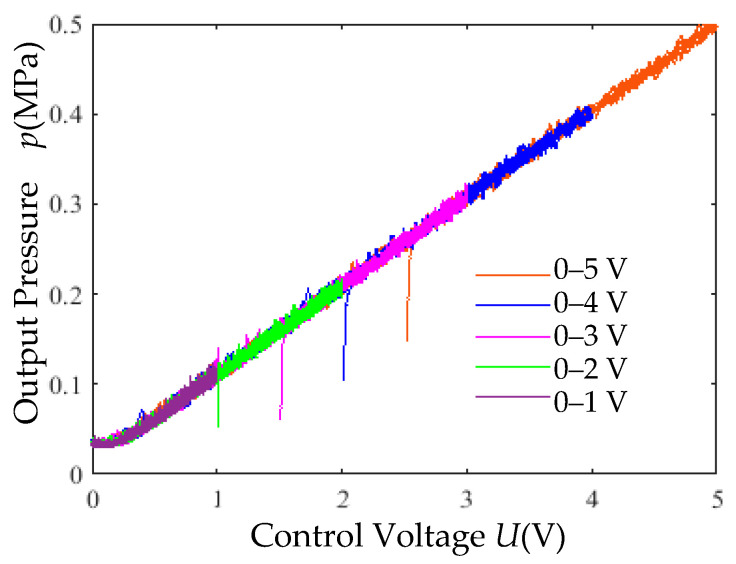
Input–output characteristic experimental curves.

**Figure 36:**
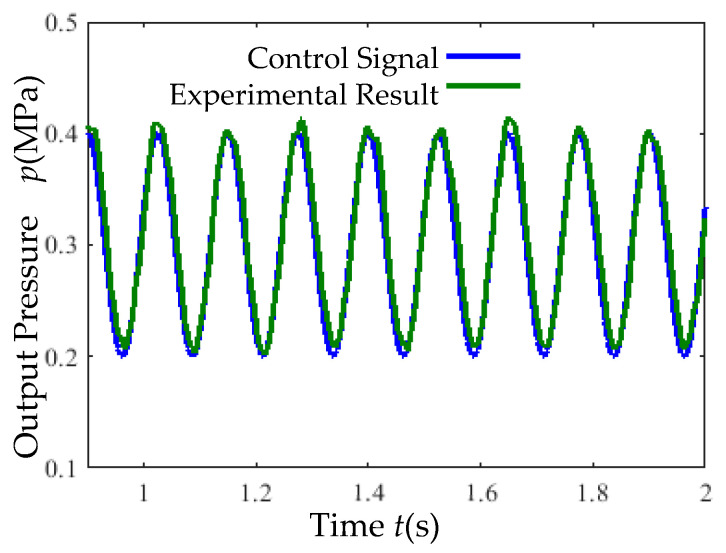
Sinusoidal response experimental curve.

**Figure 37:**
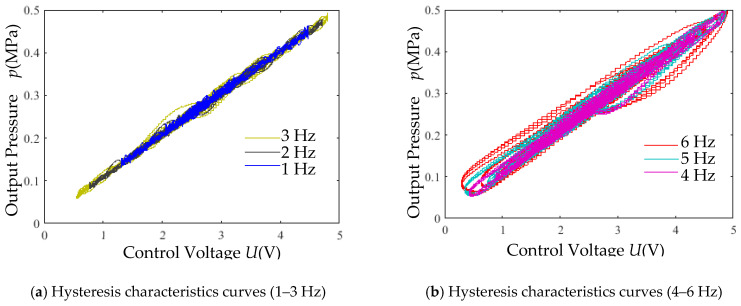
Hysteresis characteristic experimental curves.

(1) Open-Loop Hysteresis Test: Under a supply pressure of 0.7 MPa, a triangular wave control voltage signal (0–12 V, 4 s period) was applied. The output pressure *p* versus control voltage *U* relationship is shown in Figure 33.

(2) Closed-Loop Step Response Test: With the supply pressure fixed at 0.7 MPa, the step response of the incremental PID-controlled system was experimentally validated, as depicted in Figure 34.

(3) Input–Output Linearity Test: Under a 0.7 MPa supply pressure, triangular wave signals with varying amplitudes (4 s period) were input. The linearity of the proportional valve’s input–output characteristics is illustrated in Figure 35.

(4) Sinusoidal Tracking Test: A sinusoidal signal (8 Hz) was applied under a 0.7 MPa supply pressure. The output pressure tracking performance is presented in Figure 36.

(5) Hysteresis Test: Using a 0.7 Mpa supply pressure and exponential decay signals (5 V amplitude, varying frequencies), the output pressure hysteresis characteristics are plotted against the control voltage in Figure 37.

The experimental results were compared with simulation results, validating the effectiveness of the simulation model. The key findings include the following:

Dead zones and saturation zones exist in the proportional valve (Figure 33). These phenomena are attributed to the electrothermal actuation mechanism. When a control voltage is applied, Joule heating occurs in the resistor, and heat accumulation gradually opens the Po port via thermal expansion. The delayed thermal response creates dead zones. Conversely, saturation arises during voltage reduction or abrupt cutoff, where the Po port’s closure depends on the rate of heat dissipation. Slow dissipation under decreasing voltage leads to saturation.

Hysteresis is observed in the proportional valve (Figure 33). This is due to the unidirectional actuation mechanism: pressure increase requires active voltage input (Ps open, Po closed), while pressure decrease relies on passive mechanical recovery (Ps closed, Po open) driven by the lever and actuator’s restoring force without voltage input.

Rapid response capability (Figure 34). Under a supply pressure of 0.7 MPa, the step response rise time is less than 40 ms (4–4.04 s on the x-axis in figure), demonstrating rapid response speed and validating the effectiveness of the simulation results.

High linearity (Figure 35). The valve exhibits a quasi-linear control range of 30–60%.

Superior dynamic performance (Figure 36). The output pressure closely tracks an 8 Hz sinusoidal control signal with a maximum error of 0.015 MPa.

Frequency-dependent hysteresis expansion (Figure 37). Irregular hysteresis loops gradually enlarge when the input signal frequency exceeds 4 Hz.

Experimental results demonstrate that the novel proportional valve, which replaces traditional proportional solenoids with the silicon microfluidic chip as an electro-mechanical converter, achieves dynamic performance comparable to that of conventional valves (e.g., step response time ≤ 50 ms, matching FESTO’s MPYE series). Notably, the valve’s compact size (10.8 mm × 4.83 mm × 2.225 mm) significantly reduces manufacturing costs while maintaining industrial-grade precision.

## 5. Conclusions

This study demonstrates a MEMS-based pneumatic proportional pressure valve integrating a V-shaped electrothermal microactuator within a silicon microfluidic chip, addressing the limitations of traditional solenoid-driven systems in miniaturization and low-pressure precision. Key innovations include the following:

(1) Geometry-Optimized Actuator Design: A first-order transfer function was established and validated via COMSOL simulations, revealing that variable cross-section ribs reduce thermal stress by 9% while enhancing displacement. Systematic parametric analysis identified rib span length as the dominant factor for time constant reduction (64% decrease at *L* = 2.5 mm).

(2) Dual-Chip Parallelization: Dual-chip configurations achieved scalable flow capacity (5 L/min at 0.69 MPa) without sacrificing compactness, a breakthrough for industrial applications requiring both miniaturization and high flow rates.

(3) Dynamic Performance Validation: Experimental tests demonstrated rapid step response (<40 ms), low hysteresis (<5% at 1 Hz), and precise sinusoidal tracking (8 Hz, error <0.015 MPa), supported by a closed-loop PID control system.

This work bridges MEMS fabrication with pneumatic control theory, offering a transformative framework for compact, high-precision fluidic systems. Future research will focus on multi-chip arrays for high-flow scenarios and advanced control algorithms to address nonlinear hysteresis at higher frequencies.

## Figures and Tables

**Table 1:** Partial material and geometric parameters of the V-shaped electrothermal microactuator.

Property	Symbol/Unit	Value
Young’s modulus	*E*/GPa	170
Poisson’s ratio	*ν*/1	0.28
Relative permittivity	*C*/1	11.7
Thermal conductivity	*k*/W·mm^−1^·K^−1^	160
Electrical conductivity	*σ*/S·m^−1^	7518.8
Density	*ρ*/kg·m^−3^	2330
Reference resistivity	*ρ*_0_/Ω·m	16.92 × 10^−6^
Reference temperature	*T*_ref_/K	298
Rib span length	*L*/mm	5
Rib width	*D*/mm	0.12
Rib thickness	*H*/mm	0.75
Rib inclination angle	*θ*/°	3

**Table 2:** Output displacement under varying rib number and cross-section.

Number of Ribs	Cross-Section	Actuator Displacement	Lever Displacement
1	Variable	7.70	87.71
2	Variable	10.38	117.10
3	Variable	11.44	128.80
4	Variable	11.70	131.60
4	Constant	10.70	112.40

**Table 3:** Effect of rib number on static gain (*ζ*) and time constant (*τ*).

Number of Ribs	Static Gain *ζ*	Time Constant *τ*
1	10.732	0.030955
2	14.537	0.031523
3	16.0443	0.031774
4	16.407	0.032452

**Table 4:** Effect of rib cross-section on static gain (*ζ*) and time constant (*τ*).

Cross-Section	Static Gain *ζ*	Time Constant *τ*
Variable	16.407	0.032452
Constant	15.3452	0.028116

**Table 5:** Effect of rib inclination angle on static gain (*ζ*) and time constant (*τ*).

Rib Inclination Angle *θ* (°)	Static Gain *ζ*	Time Constant *τ*
1°	8.5233	0.027728
2°	13.2706	0.028576
3°	15.2221	0.028399
4°	15.3452	0.028116
5°	14.5783	0.028985
6°	13.3682	0.027278
7°	12.1444	0.027898
8°	11.9599	0.029099
9°	11.0318	0.029187
10°	10.1478	0.028741

**Table 6:** Effect of rib width on static gain (*ζ*) and time constant (*τ*).

Rib Width *D* (mm)	Static Gain *ζ*	Time Constant *τ*
0.090	16.7149	0.026304
0.105	16.1240	0.028097
0.120	15.2221	0.028399
0.135	14.2044	0.028274
0.150	13.1383	0.028924

**Table 7:** Effect of rib span length on static gain (*ζ*) and time constant (*τ*).

Rib Span Length *L* (mm)	Static Gain *ζ*	Time Constant *τ*
2.5	4.8145	0.010242
3.3	7.8827	0.015705
5.0	15.2221	0.028399

**Table 8:** Effect of rib thickness on static gain (*ζ*) and time constant (*τ*).

Rib Thickness *H* (mm)	Static Gain *ζ*	Time Constant *τ*
0.1875	15.8065	0.028127
0.375	15.9033	0.028163
0.75	15.2221	0.028399
1.125	14.3849	0.028000
1.50	13.5004	0.028970

## Data Availability

The original contributions presented in the study are included in the article; further inquiries can be directed to the corresponding author.
